# Attraction of Adults of *Cyclocephala lunulata* and *Cyclocephala barrerai* (Coleoptera: Scarabaeoidea: Melolonthidae) towards Bacteria Volatiles Isolated from Their Genital Chambers

**DOI:** 10.3390/molecules25194430

**Published:** 2020-09-27

**Authors:** Abraham Sanchez-Cruz, Norma Robledo, María Rosete-Enríquez, Angel A. Romero-López

**Affiliations:** 1 Laboratorio de Ecología Química de Insectos, Centro de Desarrollo de Productos Bióticos, Instituto Politécnico Nacional. Carretera Yautepec-Jojutla, Km. 6, calle CEPROBI No. 8, Col. San Isidro, Yautepec, Morelos C.P. 62731, Mexico; asanchezc1700@alumno.ipn.mx; 2 Laboratorio de Macromoléculas, Facultad de Ciencias Biológicas, Benemérita Universidad Autónoma de Puebla, Boulevard Capitán Carlos Camacho Espíritu, Edificio 112-A, Ciudad Universitaria, Col. Jardines de San Manuel, Puebla C. P. 72570, Mexico; maria.rosetee@correo.buap.mx; 3 Laboratorio de Infoquímicos y Otros compuestos Bióticos, Facultad de Ciencias Biológicas, Benemérita Universidad Autónoma de Puebla, Boulevard Capitán Carlos Camacho Espíritu, Edificio 112-A, Ciudad Universitaria, Col. Jardines de San Manuel, Puebla C. P. 72570, Mexico

**Keywords:** *Cyclocephala*, microbial natural products discovery, volatile organic compounds from bacteria, attraction, bioassays, EAG

## Abstract

In the study of the chemical communication of adults of the Melolonthidae family, bacteria have been observed in the epithelium of the genital chamber; possibly, bacteria are involved in the production of sex attractants in their hosts. Therefore, it is important to identify the volatile organic compounds from bacteria (VOCsB) released by these microorganisms and study the biological activity stimulated by VOBCs in adults of Melolonthidae. In this study, bacteria were isolated from the genital chamber of *Cyclocephala lunulata* and *Cyclocephala barrerai*, from which VOCsB were extracted using static headspace solid-phase microextraction (SHS-SPME) and dynamic headspace Super Q solid-phase extraction (DHS-SPE) and analyzed using gas chromatography-mass spectrometry. The effect of VOCsB on the hosts and conspecifics was evaluated utilizing an olfactometer and electroantennography (EAG). Two species of Enterobacteria were isolated from the genital chamber of each female species, and VOCsB derived from sulfur-containing compounds, alcohols, esters, and fatty acids were identified. An attraction response was observed in olfactometry studies, and antennal responses to VOCsB were confirmed in EAG bioassays. With these results, new perspectives on the relationship between these beetles and their bacteria emerge, in addition to establishing a basis for management programs in the future.

## 1. Introduction

Several genera of the Melolonthidae family as phytophages are pests of various crops [1,2]; among these is the genus *Cyclocephala* [3], which has been the less-studied of the family. Members of this genus are widely distributed across the Americas [4], and species such as *Cyclocephala lunulata* and *Cyclocephala barrerai* are important pests of diverse plant species like strawberry, guava, and ornamental pastures [2,5,6]. For the monitoring and management of these beetle species, studies have been carried out to take advantage of the substances involved in intraspecific interactions [7,8,9,10], focusing on volatile organic compounds involved in attracting a partner for copulation, such as sex pheromones [11,12]. Bacteria have been observed in the reproductive system of females of Melolonthidae, specifically in the accessory glands and the genital chamber-structures associated with sex pheromones production [10,11,12,13,14]. Based on this evidence, it has been suggested that bacteria participate in the production of these chemicals, such as in the case of *Morganella morganii* found in the accessory glands of female individuals of melolonthid *Costelytra zealandica* beetles. These microorganisms produce volatile organic compounds from bacteria (VOCsB) such as phenol, which has been reported as a component of the sex pheromone of *C. zealandica* [15,16]. The presence of the enterobacteria *Klebsiella oxytoca* and *Klebsiella michiganensis* have also been recorded in the genital chamber of *Phyllophaga obsoleta*, where a similar relationship may occur [17]. These relationships have not been studied in *Cyclocephala* (Coleoptera: Scarabaeoidea: Melolonthidae) [3].

Bacterial growth conditions, such as culture media, influence VOCsB diversity and abundance, so it is not appropriate to use general culture media to carry out studies of the production of VOCsB. Bacteria must be cultivated in specific culture media [18,19,20] containing 10% glucose and sulfur compounds, as the triple-sugar medium iron (TSI) allows the synthesis of a greater diversity of VOCsB [21]. In the extraction of VOCsB, static headspace solid-phase microextraction (SHS-SPME) has been used to capture these compounds; due to the sensitivity and how they adapt to the bacterial culture, the fibers that have been used in various studies are those of polydimethylsiloxane (PDMS), polyacrylate (PA), and/or divinylbenzene (DVB) for VOCsB of Enterobacteriaceae [22,23,24]. However, SHS-SPME is a destructive test, which limits its use in bioassays, so dynamic headspace Super Q solid-phase extraction (DHS-SPE) [25,26] is an alternative to obtain samples that can be tested in attraction bioassays; the two forms of extraction are followed by VOCsB identification by gas chromatographic mass-spectrometry (GC-MS) [19,24].

VOCsB, emitted by insect bacteria, play an important role in host chemical communication [27,28,29]. VOCsB identified in these interactions are hydrocarbons, ketones, alcohols, derivatives with nitrogen or sulfur, and terpenes [19,20]; these are involved in the chemical communication of various groups of insects [30,31]. In bioassays (olfactometer and flight tunnel), it has been observed that VOCsB may attract a single sex (sex attractant) or males and females (aggregation attractant) [32,33,34,35,36,37,38,39,40]. Even though the attraction tests have served to know the biological response of insects to VOCsB, the antennal response they have towards VOCsB is unknown; tests such as electroantennography (EAG) are fundamental to link the behavioral response with the stimulation of the olfactory receptors [38,40,41].

Therefore, this study aimed at isolating and identifying bacteria from the genital chamber of female individuals of *C. lunulata* and *C. barrerai* to capture and identify the VOCsB they emit, in addition to studying the biological activity of these compounds in the insect hosts. This knowledge could contribute to the future management of these pests where VOCsB may be an important element.

## 2. Results and Discussion

### 2.1. Bacteria Isolated from the Genital Chamber of Cyclocephala

Two culturable bacterial strains were isolated from the genital chamber of *C. lunulata* that were identified as the *Klebsiella* sp. (GenBank accession number MT239565) and *M. morganii* (GenBank accession number MT239566). Two strains identified as *K. oxytoca* (GenBank accession number MG652605) and *Citrobacter freundii* (GenBank accession number MG652606) were isolated from the females of *C. barrerai*; the strains that demonstrated the closest matches with the NCBI database are presented in Table 1. 

These bacteria grow in aerobic conditions using an enriched medium Luria Bertani (LB); the colonies are morphologically similar (Table 2) but show differences in the color and reflected light. Regarding individual characteristics, all are Gram-negative bacillus.

This is the first study of the activity of VOCsB in members of Melolonthidae from Mexico in terms of the isolation of bacteria from the genital chamber, identification of VOCsB emitted from it, and the confirmation of biological activity. The isolation and identification of microorganisms in these species of *Cyclocephala* are the first recorded for this genus and the third report for Melolonthidae [16,17]; thus, this work provides more information on the diversity of microorganisms associated with the reproductive system of these beetles. The bacterial diversity of the genital chamber of *C. lunulata* and *C. barrerai* are like each other and to that reported in other insect species [29,34,42]. The hypothesis is that bacteria can be acquired through food mainly during the larval state, and upon reaching the genital epithelium, they adapt, as that provides them with the conditions for their survival [34,36]. The host does not depend on a sole bacterial species for chemical communication, and thus, in case of losing a member of the bacterial community, the host can replace it [35]. Concerning the VOCsB produced by the genus *Klebsiella* colonies, the emission of indole is characteristic [43,44]. The genome of *K. oxytoca* suggests that this microorganism may emit short-chain alcohols [45]; the emission of 3-methyl butane-1-ol in *K. oxytoca* is the first report with an amino acid-rich culture medium [24].

### 2.2. Identification of VOCsB

**Figure 1:**
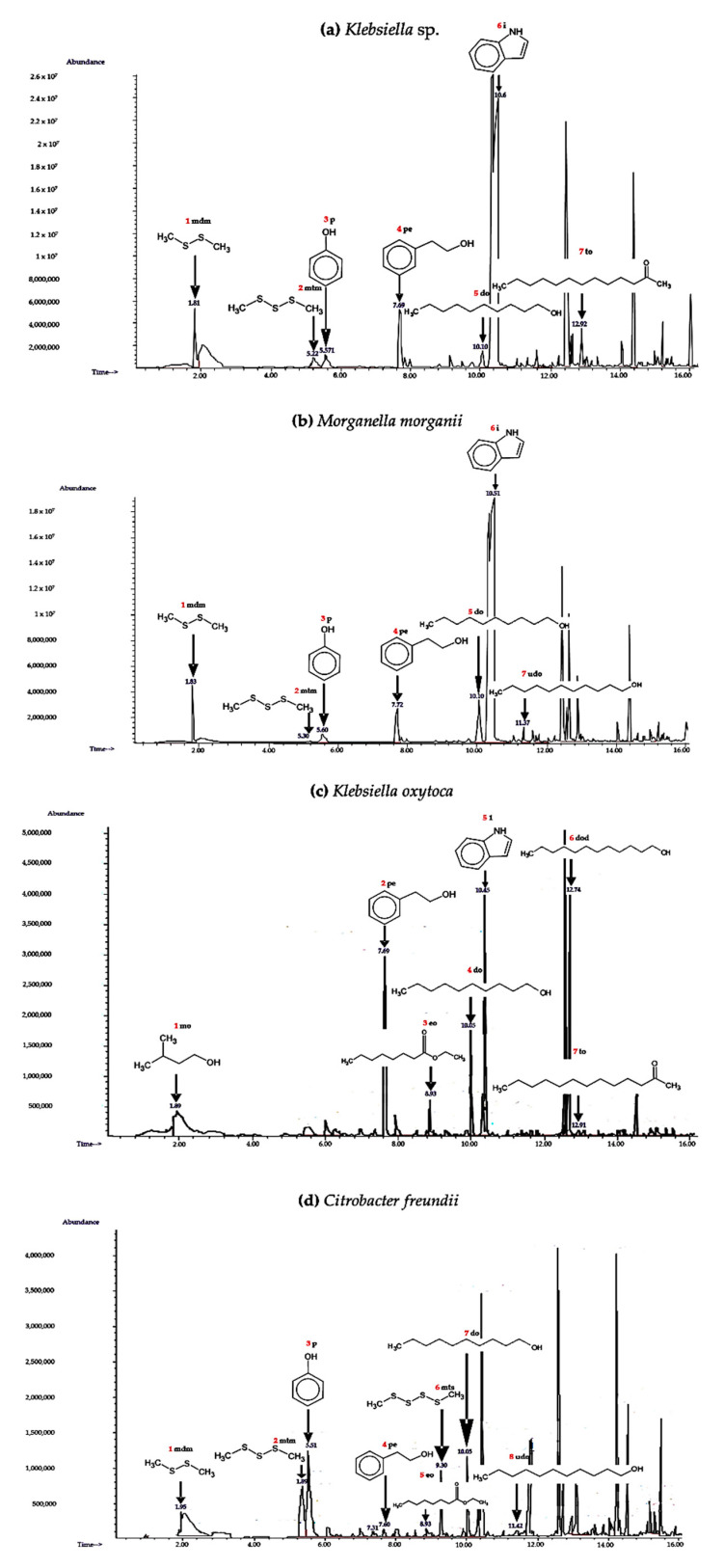
Representative total ion chromatogram and chemical structure of the volatile organic compounds from bacteria (VOCsB) from bacteria isolated in the genital chamber of *Cyclocephala lunulata* (**a**,**b**) and *Cyclocephala barrerai* (**c**,**d**). Red color: peak number and blue: retention time (minutes). (Methyl disulfanyl) methane (mdm), (methyl trisulfanyl) methane (mtm), phenol (p), 2-phenylethanol (pe), decan-1-ol (do), indole (i), tridecan-2-one (to), undecan-1-ol (vdo), 3-methylbutan-1-ol (mo), dodecan-1-ol (dod), and (methyltetrasulfanyl) methane (mts).

VOCsB recovered by SHS-SPME (Table 3), the total ion chromatogram representative of the four bacteria, are shown in Figure 1; the peaks are numbered relative to those indicated in Table 3. For both *Klebsiella* sp. and *M. morganii* bacterial strains isolated from *C. lunulata,* they were comprised of similar VOCsB derived from sulfur and large-chain alcohols, in addition to aromatic alcohols and indole, with the only difference that *Klebsiella* sp. emitted 2-tridecanone and *M. morganii* emitted 1-undecanol. For the bacterial strains isolated from *C. barrerai,* short-chain alcohol, aromatic alcohol, indole, and two fatty acid esters were recorded in *K. oxytoca*; this was the species that emitted the least number of VOCsB, in contrast to *Ci. Freundii*, where thirteen compounds were identified —three derived from sulfur, two large-chain alcohols, two aromatic alcohols, a carboxylic acid, and three fatty acid esters. Aromatic and sulfur-derived alcohols were identified in the compounds captured by DHS-SPE from *C. lunulata*, *Klebsiella* sp*.,* and *C. barrerai;* however, the *K. oxytoca* strain was distinct, since only 2-phenyl ethanol was identified. 

The genome of *K. oxytoca* suggests that this microorganism may emit short-chain alcohols [45]. The emission of aromatic alcohols is common in bacteria of the family Enterobacteriaceae [18] to which *Cyclocephala* bacteria belong, as well as the bacteria isolated from other Melolonthidae previously studied [16,17]. The emission of 3-methyl butane-1-ol in *K. oxytoca* is the first report with an amino acid-rich culture medium [24]. These aromatic alcohols, in general, are very attractive to insects in behavioral bioassays [42,55]. There is a relationship between the species that can metabolize compounds derived from sulfur and phenol; VOCsB that contain sulfur are related to the bacterial metabolism of phenol [21], such as in the case of *K. oxytoca* that did not produce phenol nor sulfur derivatives. The production of phenol is related to the attraction of conspecifics to the insects harboring the emitting bacteria; this compound is present in the pheromones of several Melolonthidae [10,41,56,57,58], including *Cyclocephala* sp. [59]

### 2.3. Insect Attraction to VOCsB 

**Figure 2:**
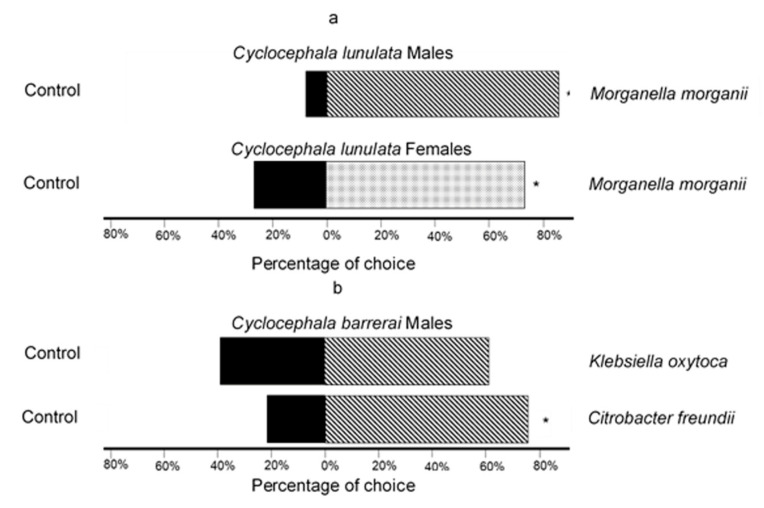
Attraction of *Cyclocephala lunulata* and *Cyclocephala barrerai* towards VOCsB from the bacteria resident of the genital chamber of females. Asterisks indicate significant differences in χ^2^ test and *p* > 0.05. (**a**) Males (n = 10) and females (n = 19) of *C. lunulata* attracted to VOCsB from *Morganella morganii*; (**b**) Males of *C. barrerai* (n = 18) attracted to VOCsB from *Klebsiella oxytoca* and *Citrobacter freundii*.

In *C. lunulata* (Figure 2a), 90% of males and 73.6% of females selected the VOCsB emitted by *M. morganii* rather than those from the control; both results were statistically significant: χ^2^ = 6.40, *p* = 0.01, degrees of freedom (df) = 9, n = 10, and χ^2^ = 4.36, *p* = 0.03, df = 18, n = 19, respectively. This demonstrates that the VOCsB from *M. morganii* attract both sexes. The results regarding attraction to the VOCsB obtained from *Klebsiella* sp. were inconclusive, and therefore, studies are still in progress. In *C. barrerai* (Figure 2b), 77.7% of males significantly preferred the compounds emitted by *Ci. freundii* rather than those of the control (χ^2^ = 5.56, *p* = 0.01, df = 17, n = 18). In regard to the VOCsB emitted by *K. oxytoca*, 61% of male individuals preferred these over the control; however, this result was not statistically significant (χ^2^ = 0.89, *p* = 0.34, df = 17, n = 18). There were no attraction tests for females of this species due to the low number of female insects captured.

**Figure 3:**
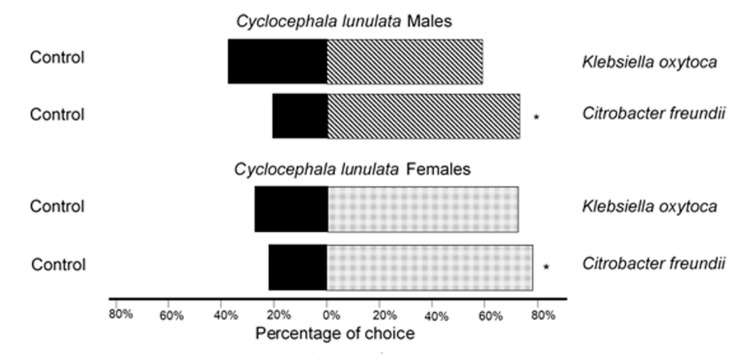
Attraction of females (n = 18) and males (n = 18) of *Cyclocephala lunulata* towards the VOCsB of the bacteria resident of the genital chamber of females of *Cyclocephala barrerai*. The asterisk indicates significant differences in χ^2^ test, and *p* > 0.05).

In tests on the attraction of *C. lunulata* to bacteria from *C. barrerai* (Figure 3), 77.7% of males selected the VOCsB emitted by *Ci. freundii* rather than those from the control (χ^2^ = 5.56, *p* = 0.01, df = 17, n = 18), 61% of males selected the VOCsB emitted by *K. oxytoca* rather than those from the control (χ^2^ = 0.89, *p* = 0.34, df = 17, n = 18). For the females of *C. lunulata*, 77.7% selected the VOCsB emitted by *Ci. freundii* rather than those from the control (χ^2^ = 5.56, *p* = 0.01, df = 17, n = 18), and 73% of females selected the VOCsB emitted by *K. oxytoca*; however, this result was not statistically significant (χ^2^ = 3.56, *p* = 0.59, df = 17, n = 18).

Although is not possible the compounds are part of the pheromones, our results confirm that VOCsB act as attractants [7], because adults of *C. lunulata* responded to VOCsB obtained from the bacteria isolated from *C. barrerai*; in particular, to those that had 2-phenyl ethanol, phenol, and sulfur derivatives. In this work, it was observed that *C. lunulata* and *C. barrerai* display attraction behaviors to VOCsB in the bioassays, walking against the wind towards the source of emission [60,61].

### 2.4. Test of EAG

**Figure 4:**
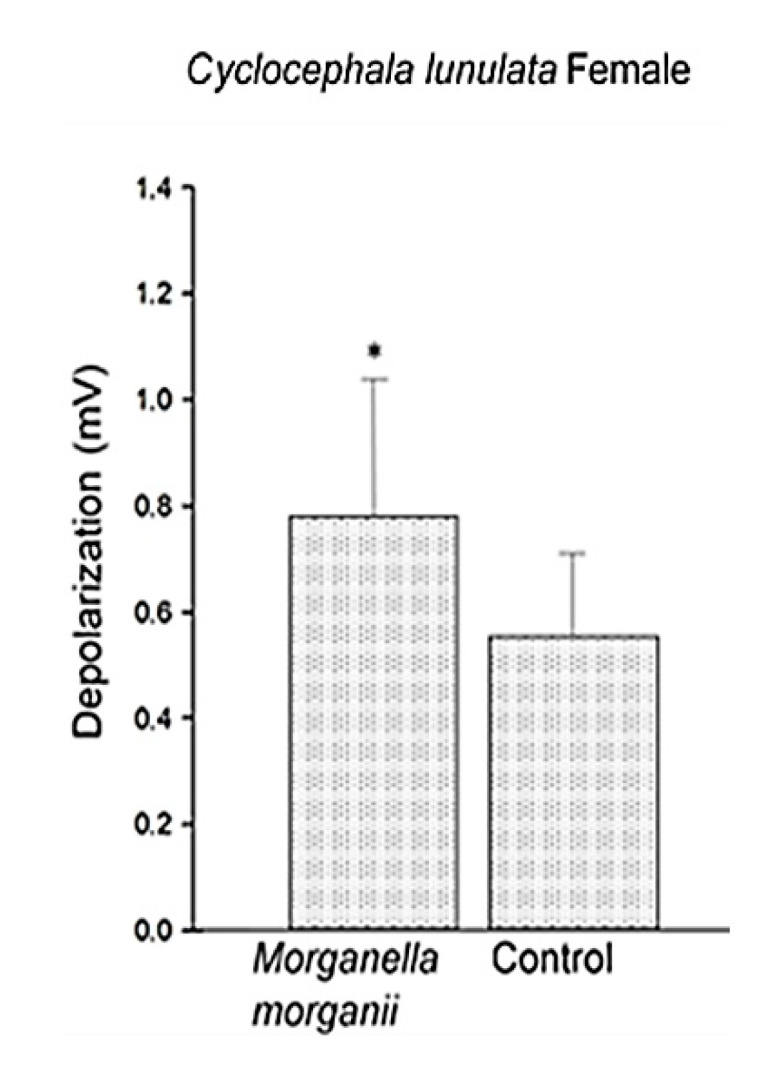
Antennal depolarization of *Cyclocephala lunulata* at VOCsB of *Morganella morganii*. The asterisk indicates significant differences in the paired *t*-test (n = 11, *p* > 0.05).

In the EAG bioassays, the antennae of female individuals of *C. lunulata* to the VOCsB of *M. morganii* (Figure 4) presented a statistically significant greater response to VOCsB than to the control (*t* = 2.80, n = 11, *p* = 0.01).

**Figure 5:**
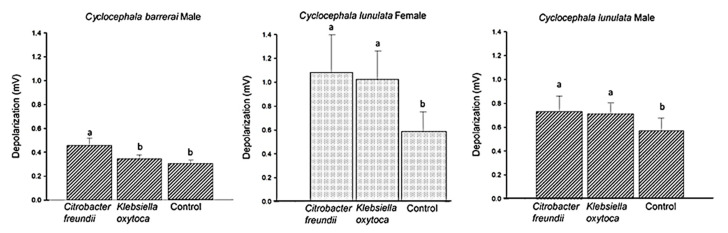
Antennal depolarization of *Cyclocephala barrerai* and *Cyclocephala lunulata* at VOCsB of *Citrobacter freundii* and *Klebsiella oxytoca*. Different letters indicate statistically different values by ANOVA Repeat Measures (RM) (Fisher Least significant difference (LSD), n = 12, *p* > 0.05).

From the antennal response to VOCsB of *Ci. freundii* and *K. oxytoca* (Figure 5), the antennae of male *C. barrerai* presented a statistically significant greater response to VOCsB than to the control (F = 7.19, df = 9, *p* < 0.01). In *C. lunulata*, the females presented a statistically significant greater response to VOCsB than to the control (F = 5.272, df = 11, *p* = 0.01) and males (F = 4.022, df = 10, *p* = 0.03).

In view of the above, the EAG tests revealed that those compounds produced antennal responses where phenol showed the greatest effects. EAG tests are important to ensure that the insect recognizes the substance to which it is exposed [62]. The attraction of beetles to VOCsB has been widely studied; however, the study of the antennal response has not, whereas EAG studies have been carried out for pheromones, allowing to relate attraction and recognition in the insect [7]. In this study, it was observed that VOCsB caused antennal depolarization, particularly those containing aromatic alcohols, such as phenol and 2-phenylethanol.

The attraction of both male and female individuals of *C. lunulata* towards several of the VOCsB indicates that these are aggregation attractants [62,63]. Aggregation has been cited for species from Dynastinae; although the production of these chemical substances has been primarily associated with males [37,39,64,65,66,67,68,69], there are reports of female beetles emitting this type of pheromone as well [70,71]. In this study, the females of *Cyclocephala* produced the compounds, possibly with the assistance of bacteria associated with their genital chamber, and this coincides with other studies reporting that VOCsB act as aggregation attractants for their insect hosts [35,36]. In other studies, the VOCsB of *Bactrocera zonata* Saunders attract a greater number of females [18], and the VOCsB from the intestinal microbiota of *Dendroctonus ponderosae* Hopkins provoke aggregation for the mass invasion of trees [72].

The emission of alcohols by bacteria from the genital chamber of *C. lunulata* could also serve a defensive role against pathogenic microorganisms, in addition to their function in insect chemical communication. Alcohol derivatives such as phenol and, particularly, long-chain alcohols are usually produced by members of Enterobacteriaceae [25], and they inhibit the growth and development of Gram-positive bacteria, fungi [2,73], and the most representative species of insect pathogens [74]. This could explain why, until now, all bacteria isolated from the female reproductive apparatus of Melolonthidae beetles belonged to Enterobacteriaceae. For instance, the intestinal bacteria of *Schistocerca gregaria* [35] act primarily as a form of defense and participate in the synthesis of food and secondarily function in the chemical communication of the host. Therefore, bacteria from different species are metabolically similar and emit similar compounds [18]. 

## 3. Materials and Methods 

### 3.1. Insects

The collection of adult specimens was conducted during the periods April-June 2017, April-June 2018, and March-July 2019. The *C. lunulata* adults were collected in green spaces within the municipalities of Yautepec (18°53′10.5″ N 99°04′17.9″ W and Cuautla, Morelos (18°49′51.5″ N 98°56′40.1″ W), Mexico between 23:00 and 24:00 h, and the adults of *C. barrerai* were collected in the green spaces of the ″Benemérita Universidad Autónoma de Puebla″ (BUAP) (19°00′17.1″ N 98°12′08.5″ W) and in “La Presa” Park (18°58’26.0″ N, 98°14’46.4″ W), Puebla, Mexico between 20:00 and 21:00 h. Insects were identified using the keys created by Morón [75]. Each species was separated according to sex, and groups of ten individuals were placed in 1-L bottles with 50% soil and organic material; every third day, the beetles were fed on *Psidium* sp. fruit, and the soil was moistened with water. The beetles were left for at least five days without copulation for the subsequent experiments. 

### 3.2. Microorganisms of the Female Reproductive System

Based on the technique proposed by Rosete-Enríquez and Romero-López [17], bacteria were extracted from the genital chamber of 10 females of *C. lunulata* and *C. barrerai*. The colony-forming units of each bacterial strain were cultivated to describe their colonial morphology and identifying bacterial morphology using Gram staining. 

Genomic DNA was extracted and purified according to the Wizard^®^ Genomic DNA Purification Kit (Promega, Madison, WI, USA). The amplification of two segments of the 16S rRNA gene was performed by a polymerase chain reaction (PCR) based on the protocol proposed by Rosete-Enríquez and Romero-López [17]. Briefly, two reactions carried out using 10 ng of genomic DNA, 200-μM deoxynucleotide solution mix (dNTPs), 1 μM of each pair of upper and lower primers, 2 or 1.5-mM MgCl_2_, 1.25 units of PlatimunTM Taq DNA polymerase, 1X PCR buffer (Invitrogen, Carlsbad, CA, USA), and DNase free water to make 20 μL. The sequence of primers for the first reaction was 8F 5′ AGAGTTTGATCCTGGCTCAG 3′ and 907R 5′ CCGTCAATTCM TTTRAGTTT 3′. The second reaction was conducted with primers 533F 5′ GTGCCAGCAGCCGCGGTAA 3′ and 1496R 5′ GGTTAC.CTTGTTACGACTT 3′. Amplifications were performed in a Techne 412 thermocycler (Bibby Scientific, Staffordshire, UK) with the following cycling program: initial denaturation for 5 min at 94 °C, 35 or 30 cycles for 45 s at 94 °C, 55°C or 56 °C for 1 min, 72 °C for 1.5 min, and a final extension at 72 °C for 10 min. The molecular weight of the amplicons was determined by comparison with the migration of the 100-bp molecular marker. The PCR amplicons were purified from band cutting following the instructions of the QIAquick Gel Extraction kit (Qiagen). Once the amplicon concentration was verified, sequencing was performed using the Sanger method in the sequencing unit of the Institute of Biology of BUAP, Mexico. The sequences were edited and assembled with the free distribution software Bioedit 7.0 [76]. Genus and species identification were carried out using BLAST in the database of the National Center for Biotechnology Information (NCBI, https://blast.ncbi.nlm.nih.gov/Blast.cgi), comparing the sequence obtained from the rRNA 16s gene with reference sequences recorded in the database. The analysis of the sequences was performed by CLUSTALW method [77] alignment, followed by a calculation of genetic pairwise distances using the neighbor-joining method [78]. Estimation of variance was performed by Bootstrap with 10,000 resamples, with a model of nucleotide substitution using the Kimura 2 parameters analysis [79] and gamma distribution pattern. The matrix of the genetic distances was completed using the freely distributed software MEGA 7 (1.0 version, State College, PA, USA) [80].

### 3.3. Extraction and Identification of VOCsB

The bacterial strains were cultivated in round, glass flasks containing 50 mL of triple-sugar iron (TSI) sterile culture. The bottles were pressure-sealed using an aluminum top with a silicon septa in its center. The system was incubated 8 h at 30 °C:

1. VOCsB were captured by SHS-SPME, inserting a 65-μM PDMS/DVB fiber (Supelco, Inc., Bellefonte, PA, USA) into the bottle and maintaining the system for 16 h at 30 °C. After removing the fiber, the sample was analyzed by GC-MS. Ten repetitions were carried out for each bacterial strain in TSI; the control was a sterile TSI medium without bacteria.
2. The capture of VOCsB by DHS-SPE was performed under the aforementioned conditions for bacterial growth in the TSI medium; cultures were incubated for a period of 8 h at 30 °C. Pasteur pipette with 125 mg of Super Q 80/100 (Alltech Assoc, Inc., Deerfield, IL, USA) was connected to the upper part of the flask through the silicone septa. Once the Pasteur pipette was inserted, the experiment was maintained for 16 h at 30 °C.

The end of the incubation period, the culture system was removed, and the end of the Pasteur pipette was connected to a Welch USA double-flow pump with a constant suction flow of 500 mL/min for 1 h at 27 °C. The compounds were diluted in 2 mL of hexane (HPLC, JT Barker®, Chemical company, NJ, USA) and reconcentrated to 200 μL with a nitrogen current. An GC-MS (7890A-5975C, Agilent, Santa Clara, CA, USA), with an HP-5MS column (19091S-433, Agilent, Santa Clara, NJ, USA) (30 m, 0.250-μm internal diameter, and 0.25-μm thickness) was used for the identification of the VOCsB captured by both extraction methods. Aliquots (2 μL) of the extracts were analyzed by GC-MS; oven start temperature of 50 °C for 2 min, increased to 220 °C by 8 °C/min, and maintained for 2 min; the injection port functioned in the splitless 1:10 mode at a temperature of 250 °C [24]; the flow was 2 mL/min of hydrogen as a carrier gas. The mass spectrometer worked with electronic ionization (70 EV) in SCAN mode and a mass range of 35 to 550 Atomic Mass Unit AMU. Only the compounds that were present in at least 90% of the test samples and absent in the control were identified by their retention times, Kovats Retention Index [81], and through comparison of their mass spectra with the spectral library NIST/EPA/NIH (Software Version 2.0, Gaithersburg, MD, USA). The chemical structure is made with the software ADC/ChemSketch Freeware 2020, Canada. 

### 3.4. Bioassays

#### 3.4.1. Olfactometer 

A glass Y-tube olfactometer consisting of a central 14-cm-long tube and two 13-cm-long arms with an internal diameter of 1.5 cm was used. Each arm was connected to a 6.5-cm-long and 2.5-cm-internal diameter glass chamber, where the stimulants were placed. For the stimulus impulse, a wind flow of 500 mL/min provided by a double-flow pump (2522B-01, WELCH, Niles, IL, USA) was used, regulated by a flowmeter (Cole-Palmer, Chicago, IL, USA), at 1 L/min in the central tube. The bioassays were conducted between 20:30 h–21:30 h for *C. barrerai* and 23:00 h–1:00 h for *C. lunulata* under controlled conditions of 27 ± 1°C, 50% relative humidity, and illuminated by a 15-W red light (Philips, Shenzhen, China). The bacterial extracts were used as stimuli and the sterile medium as the control. To the stimuli, 2 μL were added on a piece of 5 × 2-mm filter paper (Whatman No. 1^®^ 2V, Merck KGaA, Darmstadt, Germany), which were placed inside each end of the olfactometer and left for 20 s to allow the volatilization of the stimulus. The test was conducted with female and male beetles that did not copulate during the previous 5-7 days. The behavior of the adults was filmed using a camera (WB800F, Samsung, Daegu, South Korea), and the response of everyone to each stimulus was recorded. A positive response was considered as an individual reaching at least halfway along the glass arm and remaining at the side that was selected for over 30 s [60]. There was a pause of 5 min between each test during which a flow of air cleaned the system to ensure any volatile remnants were removed. The arm where the stimuli were placed was alternated randomly, and only one sex was analyzed per day. Before each bioassay, the olfactometers were washed and rinsed with hexane and dried at 100 °C. The data were analyzed using a χ^2^ test and presented as the total percentage of repetitions. The video recordings were revised, and photos were obtained that were used to chart the behavior patterns presented by the individual beetles before, and then, they selected a stimulus. 

#### 3.4.2. Electroantennography

EAG responses were conducted using EAG equipment (Syntech, Kirchzarten, Germany). A recently dissected antenna of each adult was mounted between 2 silver electrodes using conductor gel (Sigma gel, SYNTECH, Spectra 360, Parker, Orange, NJ, USA). The signal generated by the antenna was transmitted to an IDAC-2 amplifier, recorded, and analyzed with software (SYNTECH EAG PRO 2.0, 2005, SYNTECH, Hilversum, The Netherlands). A constant flow of humidified pure air (0.5 L/min) provided by a pump (stimulus controller SC-55) was directed onto the antenna through a glass tube (diameter 10 mm). To present a stimulus, a pipette tip containing the stimulus of 2 μL of VOCsB extract headspace was placed on filter paper (5 × 2 mm, Whatman No. 1) was inserted through a side hole located at the midpoint of the glass tube. The outlet of the glass tube was positioned approximately 2 cm from the antenna. Humidified pure air flowed at 0.5 L/min through the pipette during stimulation. For stimulus application, 2 μL of stimuli were placed on filter paper (5 × 2 mm, Whatman No. 1). A dilution of standard eucalyptol (50 μL/mL) was used as an entrance and exit stimulus for *C. lunulata* and linalool (50 μL/mL) for *C. barrerai* to verify the basal state of the antenna. All the standards were provided by Sigma Aldrich^®^ (Toluca, Mexico). The stimuli were the VOCsB extracts and the control (extract of TSI); they were randomly placed in the arms of the olfactometer. In each experiment, the solvent was left to evaporate for 20 s, and the time interval between each stimulus was 60 s. In these bioassays, the antennal depolarization response to the solvent was subtracted from the response to the stimuli before analysis. Once obtained, the antennal responses were analyzed with the software; the data were normalized, calculating the EAG values as a relative percentage to the entrance and exit stimuli to eliminate the error caused by a loss of sensibility in the antenna.

### 3.5. Statistical Analysis

Olfactometry bioassays behavior was analyzed using χ^2^. EAG data were log-transformed and analyzed by a paired *t*-test and ANOVA Repeat Measures (RM), SigmaPlot 12.0, SYSTAT Software, Chicago, IL, USA.

## 4. Conclusions

Female *C. lunulata* and *C. barrerai* possess bacteria in their genital chamber, *C. lunulata* possesses the bacteria *M. morganii* and *Klebsiella* sp., and female *C. barrerai* possess the bacteria *K. oxytoca* and *Ci. freundii*. *Klebsiella* sp. and *M. morganii* emit VOCsB derived from sulfur; alcohols; and aromatic alcohols such as methyltrisulfanyl methane, decan-1-ol, and phenol. VOCsB of *M. morganii* cause attraction responses and antenatal responses in females and males of *C. lunulata*, functioning as aggregation attractants. *Klebsiella oxytoca* emits VOCsB such as aromatic compounds and aliphatic and aromatic alcohols, including decan-1-ol and 2-phenyletanol. In the bioassays, no significant differences were observed in the attraction responses of males of *C. barrerai* and males and females of *C. lunulata* to *K. oxytoca* VOCsB, although these compounds caused an antennal response in these beetles. Finally, *Ci. freundii* emitted a large number of sulfur-derived compounds; alcohols; and aromatic alcohols such as (methyldisulfanyl) methane, (methyltrisulfanyl) methane decan-1-ol, and phenol. These VOCsB cause the attraction of male *C. barrerai* and male and female *C. lunulata*, functioning as aggregation attractants. As VOCsB function as aggregation attractants, they could be used in mass trapping for the management of *C. lunulata*. This study is novel in terms of the identification of VOCsB and their biological activity in their hosts.

## Figures and Tables

**Table 1:** Identification of the bacterial strains isolated from the genital chamber of *Cyclocephala lunulata* and *Cyclocephala barrerai* by comparing the homologous sequences of the 16S rRNA gene.

Melolonthidae Specie	Strain	Closest Matches	Accession no.	Similarity (%)	Phylogenetic Affiliation	Host/Isolation Source
*Cyclocephala lunulata*	*Klebsiella* sp. MT239565	*Klebsiella spallanzanii* strain SPARK1058C2	MN091365.1	100	Enterobacteriaceae	*Homo sapiens* (urine)
		*Klebsiella grimontii* strain SS141	CP044527.1	100	Enterobacteriaceae	Coffee cup
		*Klebsiella oxytoca* strain 4928STDY7387762	LR607363.1	100	Enterobacteriaceae	Feces
	*Morganella morganii* MT239566	*Morganella morganii* strain FC6853	MH628232.1	100	Enterobacteriaceae	*Homo sapiens* (patient)
		Uncultured organism clone ELU0075-T355-S-NIPCRAMgANa_000386	HQ774639.1	100	Enterobacteriaceae	*Homo sapiens* (gastrointestinal)
		*Morganella morganii* subsp. morganii strain JY 16S	KR094121.1	100	Enterobacteriaceae	Medical College, Soochow University
*Cyclocephala barrerai*	*Citrobacter freundii* Cyl_Citf01 MG652605	*Citrobacter freundii* strain FDAARGOS_61	CP026045	99	Enterobacteriaceae	*Homo sapiens* (rectal swab)
		*Citrobacter freundii* strain BD	CP018810	99	Enterobacteriaceae	*Bactrocera dorsalis* (gut)
		*Citrobacter* sp. PT2B	GU458283	99	Enterobacteriaceae	*Reticulitermes speratus* (gut)
	*Klebsiella oxytoca* Cyl_Kleb02 MG652606	Uncultured bacterium	JF208909	100	Enterobacteriaceae	*Homo sapiens* (skin)
		*Klebsiella oxytoca* strain CAU9419	MF428632	99	Enterobacteriaceae	pickle
		*Klebsiella oxytoca* strain NGB-FR100	LC049195	99	Enterobacteriaceae	Root nodules of fava bean

**Table 2:** Morphological characteristics of colonial and individual bacteria isolated from the genital chamber of females of *Cyclocephala lunulata* and *Cyclocephala barrerai*.

Morphology	*Cyclocephala lunulata*		*Cyclocephala barrerai*	
	*Klebsiella* sp.	*Morganella morganii*	*Klebsiella oxytoca*	*Citrobacter freundii*
Shape	Circular	Circular	Circular	Circular
Edges	Rounded	Rounded	Rounded	Rounded
Elevation	Convex	Convex	Convex	Convex
Surface	Smooth	Smooth	Smooth	Smooth
Consistency	Creamy	Creamy	Creamy	Creamy
Pigmentation	Beige	Yellow	Beige	Yellow
Transmitted light	Translucent	Translucent	Translucent	Translucent
Optical property	Iridescent	Brilliant	Iridescent	Brilliant
Size (mm)	1	1	1−2	1
Individual morphology	bacillus	bacillus	bacillus	bacillus
Gram’s reaction	Negative	Negative	Negative	Negative

**Table 3:** VOCsB from the *Klebsiella* sp. captured by SHS-SPME and DHS-SPE.

Specie Bacteria	Pk No.	Extraction Technique	VOCsB	KRI	Ref. KRI	Characteristic EI ions
*Klebsiella* sp.	1	SHS-SPME	(methyl disulfanyl) methane	691	735 [46]	45, 61, 69, 79,83, 91, 94 (M+)
	2	SHS-SPME	(methyl trisulfanyl) methane	966	961 [46]	64, 79, 83, 111, 113, 126 (M+)
	3	SHS-SPME	phenol	977	980 [47]	40, 55, 66, 74, 94 (M+)
	4	SHS-SPME	2-phenylethanol	1087	1078 [48]	51, 65, 77, 91, 92, 122 (M+)
	5	SHS-SPME	decan-1-ol	1234	1256 [49]	43, 70, 83, 97, 112, 140, 158 (M+)
	6	SHS-SPME	indole	1251	1288 [50]	40, 58, 63, 74, 90, 102, 117 (M+)
	7	SHS-SPME	tridecan-2-one	1475	1474 [51]	43, 58, 71, 85, 96, 140, 198 (M+)
*Morganella morganii*	1	SHS-SPME	(methyl disulfanyl) methane	691	735 [46]	45, 61, 69, 79,83, 91, 94 (M+)
	2	SHS-SPME/DHS-SPE	(methyl trisulfanyl) methane	966	961 [46]	64, 79, 83, 111, 113, 126 (M+)
	3	SHS-SPME/DHS-SPE	phenol	977	980 [47]	40, 55, 66, 74, 94 (M+)
	4	SHS-SPME/DHS-SPE	2-phenylethanol	1087	1078 [48]	51, 65, 77, 91, 92, 122 (M+)
	5	SHS-SPME	decan-1-ol	1234	1256 [49]	43, 70, 83, 97, 112, 140, 158 (M+)
	6	SHS-SPME	indole	1251	1288 [50]	40, 58, 63, 74, 90, 102, 117 (M+)
	7	SHS-SPME	undecan-1-ol	1282	1372 [51]	55, 69, 83, 97, 111, 154, 172 (M+)
*Klebsiella oxytoca*	1	SHS-SPME	3-methylbutan-1-ol	688	734 [50]	42, 55, 57, 70, 87, 88 (M+)
	2	SHS-SPME/DHS-SPE	2-phenylethanol	1087	1078 [48]	51, 65, 77, 91, 92, 122 (M+)
	3	SHS-SPME	ethyl octanoate	1179	1192 [52]	57, 88, 127, 172 (M+)
	4	SHS-SPME	decan-1-ol	1234	1256 [49]	43, 70, 83, 97, 112, 140, 158 (M+)
	5	SHS-SPME	indole	1251	1288 [50]	40, 58, 63, 74, 90, 102, 117 (M+),
	6	SHS-SPME	dodecan-1-ol	1461	1473 [53]	69,83, 97,111, 140, 168, 186 (M+)
	7	SHS-SPME	tridecan-2-one	1475	1474 [51]	58, 71, 140, 169, 183, 198 (M+)
*Citrobacter freundii*	1	SHS-SPME	(methyldisulfanyl) methane	691	735 [46]	45, 61, 69, 79,83, 91, 94 (M+)
	2	SHS-SPME/DHS-SPE	(methyltrisulfanyl) methane	966	961 [46]	64, 79, 83, 111, 113, 126 (M+)
	3	SHS-SPME/DHS-SPE	phenol	977	980 [47]	40, 55, 66, 74, 94 (M+)
	4	SHS-SPME/DHS-SPE	2-phenylethanol	1087	1078 [48]	51, 65, 77, 91, 92, 122 (M+)
	5	SHS-SPME	ethyl octanoate	1179	1192 [52]	57, 88, 127, 143, 157, 172 (M+)
	6	SHS-SPME/DHS-SPE	(methyltetrasulfanyl) methane	1204	1210 [54]	45, 64, 79, 94, 111, 145, 158 (M+)
	7	SHS-SPME	decan-1-ol	1234	1256 [49]	43, 70, 83, 97, 112, 140, 158 (M+)
	8	SHS-SPME	undecan-1-ol	1282	1372 [51]	55, 69, 83, 97, 111, 154, 172 (M+)

Pk No (peak number), headspace-solid phase microextraction (SHS-SPME), dynamic headspace Super Q solid-phase extraction (DHS-SPE), volatile organic compounds from bacteria (VOCsB), and Kovats Retention Index (KRI), Electron Ionization (EI).
